# Supplementary material for: Learning curve and surgical outcome of robotic assisted colorectal surgery with ERAS program
Source: Sci Rep. 2022 Nov 29;12:20566. doi: 10.1038/s41598-022-24665-w (PMC9709162; doi:10.1038/s41598-022-24665-w)
Supplement: Supplementary file 2 — Supplementary Information 2. [file 41598_2022_24665_MOESM2_ESM.zip › RAL Raw data-Ñ[▒K/053/HS_ñw╝╨░O▒Kñσ.pdf]

正式摘要(電子簽章：000000208879584)<br>W52 / W52 ☐ 英文標題(注意:無英文姓名-請補病人基本檔!)	☐【讀取】憑證	

臺中榮民總醫院

出院病歷摘要

轉入號 : 轉出號 :

[redacted]

**(8)**流水編號	
**(9)**入院日期	110/01/17 (2021) 直腸外科 W52 - 145
**(10)**轉科日期	
**(11)**出院日期	110/01/24 (2021) 住院天數計 7 日

診斷

- 入院 :Rectum melanoma s/p CCRT
- 出院 :Rectum melanoma s/p CCRT s/p robotic assisted APR on 2021/01/18

主訴

- diarrhea since last October

病史

- This 53 years old female patient didn't have special systemic disease and operation history. She complained diarrhea about 7 times per day since this October. There was no bloody or tarry stool but the quality of stool was mucinous. She came to LMD for help and ulcerative tumor at 1cm above dentale line was found in anal scope. She was transferred to our hospital. Biopsy showed malignant melanoma. PET showed no obvious distal metastasis. Short course radiotherapy was started from 1/5. This time, she was admitted for surgical treatment.

體檢發現

- PS 70%
  Sigmoidoscopy was done and up to 10cm AAV, an ulcerative tumor was noted at 1cm AAV, located at 4'~6' position, s/p biopsy.
  1100113 No side effect
  - General appearance :
    - easy looking , conscious clear , without cardiopulmonary distress
    - Vital signs自動帶入date 體溫(BT) 脈搏(P) 呼吸(R) 血壓(BP) 身高(BH) 體重(BW) BMI
      20210117 35.7℃ 96次/分 18次/分 144/83mmHg 157.3cm 62.2kg 25.1
  - HEENT :
    - Eyes: no anemic conjunctiva, no icteric sclera, normal EOM
    - ENT: no otorrhea, no rhinorrhea, no erythematous mucosa, no injected tonsil, no deformity
  - Neck: supple, freely movable, trachea midline,no deformity, no engorged jugular vein , no palpable thyroid or lymph node enlargement
  - Chest:
    - Inspection: Grossly normal, symmetric and fully expansion, no accessory muscle used, no operation scar, no spider angiomata, no gynecomastia
    - Palpation: no palpable mass or nodule, no subcutaneous emphysema, no tenderness
    - Ausculation: clear breathing sound
  - Heart:
    - Percussion: normal heart size
    - Ausculation: regular heart beat, normal S1, S2, no gallop, no murmur
  - Abdomen:
    - Inspection: in
      - flat contour, symetrically, no scar, no stria, no dilated veins, no rashes
    - Ausculation: normoactive bowel sound, no abdominal bruits
    - palpation: soft, no muscle guarding or rigidity, no local tenderness, no rebounding pain, no palpable mass, no palpable pulsation

-
  - Percussion: no shifting dulllness, no hepatomegaly, no splenomagaly
-

Peripheral pulsation :	CA	BA	RA	FA	PA	PTA	DPA
Right side :	++	++	++	++	++	++	++/+++
Left side :	++	++	++	++	++	++	++/+++

- Genito-anus: no tenderness or palpable mass lesion or rectal shelf, normal external genitalia, normal anal tone, brown stool

CRS CONDITION

- Colonoscope report: Sigmoidoscopy was done and up to 10cm AAV, an ulcerative tumor was noted at 1cm AAV, located at 4'~6' position, s/p biopsy.
- CEA: 0.757 ng/ml
-
  - Lung

手術日期及方法

- 手術日期及方法20210118 1. Robotic (XI) assisted abdominal-perineal resection with end S-colostomy
  2. Intraoperative colonoscopy : (-).
  3. High ligation (+) : At IMA root. Left colic artery and IMV were also ligated via robotic endoclips.
  4. Take down from the splenic flexure : (-)
  5. Robotic endoGIA (45-4.3) X1 was applied to resected the S-colon.
  6. Proximal cut end: 8 cm
  7. Two 12 Fr. penrose drainage tube were placed from the perineal wound into the pelvic cavity.
  8. The perineal wound was closed with interrupted 2-0 Nylon and then covered with gauze.

住院治療經過

- After admission, robotic assisted APR was done on 2021/01/18. We let her try soft diet on POD2. Foley and penrose drain was removed on POD4, but Foley was re-inserted due to urinary difficulty. Under the stable condition, she was discharged on 1/24.

合併症

- nil

一般檢查

- 累積報告 :

DATE	TIME	ALB	TP	BIL.T	BIL.D	ALKP	AST	ALT	LDH	CHOL	TG	HDL-C	LDL	UA	NH3	AMY
1100117	2051			0.29			23	8								
1100119	0303	2.7		0.45			10	5								

DATE	TIME	WBC	RBC	HGB	HCT	MCV	PLT	BLST	NEUT	BAND	SEG	LYM	MON	EOS	BAS
1100117	2051	4540	3.63	7.1	25.6	70.5	268		79.0			13.4	4.6	2.6	0.4
1100118	1157			8.2											
1100119	0303	7420		8.1	28.1		246		85.6			10.4	3.6	0.1	0.3

DATE	TIME	NA	K	CL	CA	BUN	CREAT	Mg	P	CRP	ESR	PCT	LIPASE	LACTATE
1100117	2051	138	4.1		8.5	10	0.54							
1100119	0303	141	3.5		7.3	5	0.51							

DATE	TIME	GLUCOSE	PAB	TRF	ZN	CU	KETONE	CK	CKMB	PT-P	PT-C	APTT-P	APTT-C	TRO-T
1100117	2050									10.4	10.3	28.6		

- 放射線報告 :**檢驗項目 :CHEST AP VIEW (PORTABLE) 報告日期 :1100121**
  主　訴 :
  Result:
  Finding:
  > The heart size is within normal limit.
  > On nasogastric tube, CVC, and EKG leads
  > Radiopaque opacities over right upper abdomen, gallstone can't be excluded.
  > No active lung lesion
  Impression:
  As above description.

特殊檢查

病理報告

其它

出院時情況

- 3.改門診治療

出院指示

藥名	劑量	劑量單位	用法	頻次	數量	數量單位	現況	天數	單位
Ultracet tab	1	TAB	PO	Q6H	12	TAB	TH	3	天
Ferrous Gluco-B	1	TAB	PO	TIDAC	9	TAB	TH	3	天
Estazolam tab 2mg	1	TAB	PO	HS	3	TAB	TH	3	天
Ulstop tab 20mg	1	TAB	PO	BID	6	TAB	TH	3	天
Mag. Oxide tab	2	TAB	PO	TID	18	TAB	TH	3	天

出院計劃

主治醫師 2062F 蔣鋒帆 / 住院醫師 2331F 陳凱祥

正式摘要**(**電子簽章：**000000208879584)**

W52 / W52

doctorRank:B
